# Natural killer cells in non-hematopoietic malignancies

**DOI:** 10.3389/fimmu.2012.00395

**Published:** 2012-12-24

**Authors:** Mélanie Desbois, Sylvie Rusakiewicz, Clara Locher, Laurence Zitvogel, Nathalie Chaput

**Affiliations:** ^1^Institut de Cancérologie Gustave RoussyVillejuif, France; ^2^Centre d’Investigation Clinique Biothérapie 507, Institut de cancérologie Gustave RoussyVillejuif, France; ^3^Faculté de Médecine, Université Paris-SudLe Kremlin-Bicȴtre, Francexs; ^4^Institut National de la Santé et de la Recherche Médicale, U1015, Institut de cancérologie Gustave RoussyVillejuif, France

**Keywords:** NK cells, maturation stage, environment, tumors, metastases

## Abstract

Natural killer (NK) cells belong to the innate immune system and were initially described functionallywise by their spontaneous cytotoxic potential against transformed or virus-infected cells. A delicate balance between activating and inhibiting receptors regulates NK cell tolerance. A better understanding of tissue resident NK cells, of NK cell maturation stages and migration patterns has evolved allowing a thoughtful evaluation of their *modus operandi*. While evidence has been brought up for their relevance as gate keepers in some hematopoietic malignancies, the role of NK cells against progression and dissemination of solid tumors remains questionable. Hence, many studies pointed out the functional defects of the rare NK cell infiltrates found in tumor beds and the lack of efficacy of adoptively transferred NK cells in patients. However, several preclinical evidences suggest their anti-metastatic role in a variety of mouse tumor models. In the present review, we discuss NK cell functions according to their maturation stage and environmental milieu, the receptor/ligand interactions dictating tumor cell recognition and recapitulate translational studies aimed at deciphering their prognostic or predictive role against human solid malignancies.

## NK CELL FUNCTIONS ARE DICTATED BY THEIR STAGE OF MATURATION AND THE MICROENVIRONMENT

Natural killer (NK) cells can have distinct functions depending on their maturation stage and the microenvironment, rendering complex the physio-pathological role attributed to NK cells. The majority of mature NK cells circulate in the peripheral blood, but are also resident in several lymphoid and non-lymphoid organs, such as the spleen, tonsils, lymph nodes (LNs), liver, lungs, intestine, and uterus (Shi et al., 2011).

### MOUSE NK CELLS

**FIGURE 1:**
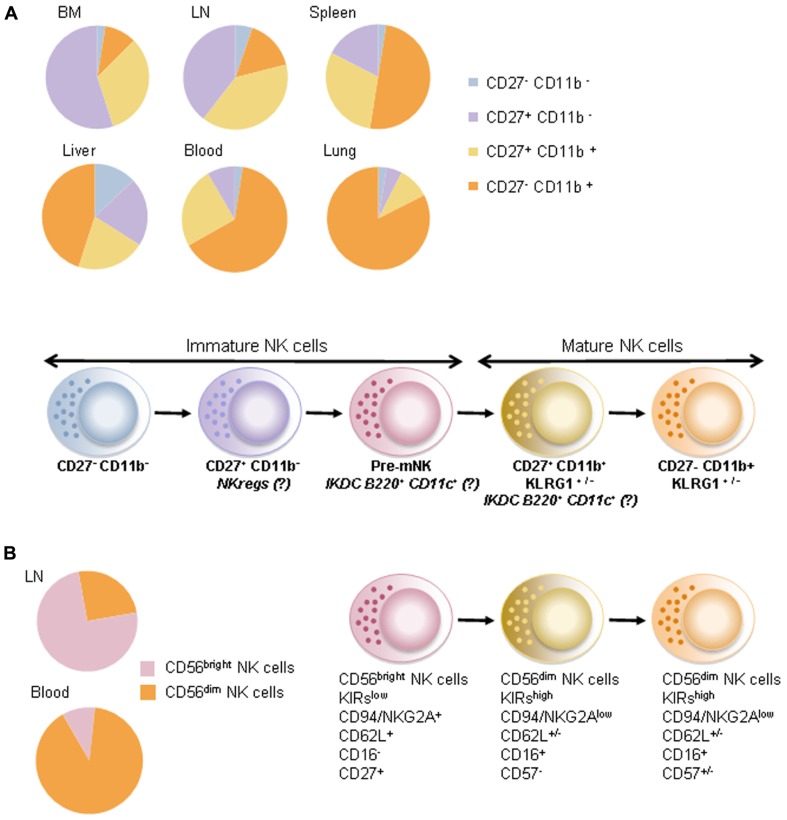
**Maturation stage of NK cells**. **(A)** In mice, the maturation stage of CD3^−^ NK1.1^+^ NKp46^+^ NK cells is defined using both CD27 and CD11b molecule expressions. Upper panel shows relative proportions of NK cells at steady state in bone marrow (BM), lymph nodes (LN), spleen, liver, blood, and lungs. Lower panel shows the differentiation of NK cells based on the expression of CD27 and CD11b. IKDCs expressing B220 and CD11c molecules would be part of a premature NK cell fraction (pre-mNK) and of a CD27-positive fraction of mature NK cells also expressing the mannose receptor (CD206). NKreg cells have an immature phenotype in that they are CD27-positive and CD11b-negative. **(B)** In humans, CD19^−^CD3^−^CD56^+^ NK cells are generally defined by high expression (bright) or intermediate (dim) expression of the CD56 molecule. As in mice, these two populations (CD56^bright^ and CD56^dim^) are enriched in separate compartments: CD56^bright^ NK cells predominate in lymph nodes while CD56^dim^ NK cells predominate in peripheral blood. Various studies suggested that NK cells expressing CD56^dim^ would be more terminally differentiated compared to NK cells expressing CD56^bright^.

In mice, the phenotype of NK cells is defined among lymphocytes as CD3^−^NK1.1^+^NKp46^+^. Mature NK (mNK) cells are identified by the expression of the integrin CD11b (Kim et al., 2002) and can be further subdivided into various subsets according to the differentiation marker CD27 (Hayakawa and Smyth, 2006). In adoptive transfer experiments, CD11b^−^ immature NK (iNK) cells pass through the CD27^+^ stage prior to reaching the terminal CD27^−^CD11b^+^ stage (Chiossone et al., 2009). CD27^+^ NK cells harbored greater cytotoxic function, proliferation capacity, and interferon-γ (IFN-γ) secretion after stimulation with interleukin (IL)-12 and/or IL-18 than the CD27^−^ expressing counterparts. CXCR3 is exclusively expressed by the CD11b^+^CD27^+^ NK cell subset, endowing cells with an active chemotaxis toward CXCR3 ligands (CXCL10, IP-10, CXCL11) and a preferred migration to lymphoid tissues (Hayakawa and Smyth, 2006). Hence, CD11b^+^CD27^−^ NK cells are considered terminally differentiated, long-lived cells, preferentially located in non-lymphoid tissues and exhibiting a functional restriction by self-major histocompatibility complexes (MHC; **Figure 1**).

A recent study described a premature NK cell subset (pre-mNK; Guimont-Desrochers et al., 2012) preceding the CD27^+^ mNK cell stage. This NK cell stage may correspond to the interferon-producing killer dendritic cells (IKDC) described in 2006 (Chan et al., 2006; Taieb et al., 2006). Indeed, Taieb et al. (2006) and others (Chan et al., 2006) described CD11c^int^ B220^+^CD49b^+^NK1.1^+^MHC-II^+^ cells sharing both antigen presentation and cytotoxic functions culminating in anti-tumor activity (Taieb et al., 2006). Guimont-Desrochers et al. (2012) showed that mNK cells fail to acquire B220, even after *in vivo* stimulation, suggesting that CD11c^int^ B220^+^ NK cells may not merely result from NK cell activation (Vosshenrich et al., 2007). They showed that, by 5 days post-transfer, CD11c^int^ B220^+^CD49b^+^ NK cells down-regulated CD11c, B220, and CD27, and acquired CD43, in agreement with functional maturation. By 25 days post-transfer, all B220^+^ NK cells had completely lost B220 expression and exhibited a CD11c^neg/low^ CD11b^+^CD27^−^ phenotype compatible with terminally differentiated NK cells. These pre-mNK cells were actively cycling, a characteristic associated with precursor cell populations (Guimont-Desrochers et al., 2012). Thus, CD27^+^ CD11b^low^ CD49b^+^ CD11c^int^ B220^+^ NK cells could be defined as pre-mNK cells, an immediate precursor to CD27^+^ CD11b^+^ NK cells. Moreover, our data pointed out that half of IKDCs were CD11b^+^. Such CD11b^+^CD27^+^IKDCs expressed the mannose receptor (CD206) accounting for ovalbumin (OVA) protein uptake and OVA-specific CD8^+^ T cell priming *in vivo*. Thus, we reported that the mature fraction of IKDCs could exert antigen-presentation capacity (Terme et al., 2009).

Interestingly, among the iNK cells (defined as CD11b^−^CD27^+^), we recently identified a distinct subset endowed with regulatory functions (that we named “NKreg”). NK cells defined as CD27^+^ CD11b^low^ c-Kit^+^ NKp46^+^ NK cells expressed molecules associated with immunosuppressive functions (such as CTLA4, Lag-3, and PDL-1). These Kit^+^ NK cells accumulated in primary and secondary lymphoid organs of tumor bearers in an IL-18-dependent fashion. Blockade of tumor-derived IL-18 markedly prevented NKreg accumulation and melanoma dissemination. Kit^+^ NK cells operated their immunosuppressive effect by directly killing immature dendritic cells (DC) and indirectly reducing the pool of peripheral mNK cells. Anti-PD1 or anti-PDL-1 neutralizing antibodies counteracted all these immunosuppressive effects. Hence, the adoptive transfer of NKreg cells promoted tumor outgrowth and metastases spreading (Terme et al., 2012). Our study is in accordance with others describing iNK cells with regulatory functions. In a mouse model of leukemia, CD49b^+^ CD90^bright^ c-Kit^dim^ NK cells expanded; compromised DC maturation and could blunt allogeneic T cell proliferation (Ebata et al., 2006). Hence, these data indicate that distinct subsets of “imNK” could exert intrinsic inhibitory functions. In addition, mNK may secrete suppressive cytokines and exert regulatory functions in pathology such as infections, autoimmunity, and transplantation. Perona-Wright et al. (2009) showed that NK cells could secrete IL-10 during the acute phase of systemic infections. NK cell-derived IL-10 hampered the expression of MHC and co-stimulatory molecules and suppressed the production of pro-inflammatory cytokines by DC (Perona-Wright et al., 2009). In autoimmune disease, NK3-like cells secreting transforming growth factor beta (TGF-β) could protect mice against type I diabetes (Zhou et al., 2007). NK cells promoted allograft tolerance (Beilke et al., 2005) and reduced graft versus host disease (GVHD) by limiting allogeneic T cell proliferation (Noval Rivas et al., 2010), presumably through eradication of myeloid DC.

Finally, NK cells from extra-medullary tissues harboring tissue-specific phenotypes suggest that the maturation can be completed in organ different from bone marrow including thymus, liver, uterus, and gut. Thymic NK cells have several peculiarities compared with “conventional” NK cells: (i) they express high levels of IL-7Rα chain (CD127) and the transcription factor GATA-3, (ii) they produce cytokines but display low natural cytotoxicity, (iii) they accumulate in LNs (Vosshenrich et al., 2006). Uterine NK cells promote vascular remodeling and influence the recruitment and activation of other leukocytes to regulate the physiology of pregnancy (Santoni et al., 2007). In the liver, bone marrow-derived NK cells migrate into the liver where they differentiate into liver-specific NK cells (Vanderkerken et al., 1993). Hepatic NK cells display potent cytotoxic capacity expressing high levels of TRAIL, granzyme B, and perforin molecules (Ochi et al., 2004; Takeda et al., 2005). A recent study has shown that CD11c^+^ NK cells could play a major role during adenoviral hepatitis (Burt et al., 2008), these CD11c^+^ NK cells gained a modest antigen-presenting cell capacity thus functionally resembling to NKDCs (Pillarisetty et al., 2005; Plitas et al., 2007) or IKDCs (Taieb et al., 2006). Finally, CXCL16 (the ligand for CXCR6) is constitutively secreted by the liver sinusoidal endothelium leading to the accumulation of CXCR6^+^ NK cells that represent a consistent fraction of liver NK cells. CXCR6^+^ NK cells are drastically reduced in CXCR6 knockout mice. CXCR6-expressing NK cell has been suggested to develop an adaptive-like immunity, memory NK cell development depend on CXCR6 expression by NK cells a chemokine receptor required for the maintenance of these memory NK cells (Paust et al., 2010). In the mouse intestine, two subsets of NKp46^+^ cells are present in the gut (Satoh-Takayama et al., 2008). Conventional NK cells secrete IFN-γ and lack expression of RORγt, whereas RORγt^+^ NKp46^+^ cells secrete IL-22 (also called “NK22”) following stimulation with IL-23 and do not require IL-15 for their differentiation (Luci et al., 2009). IL-15-dependent NKp46^+^ NK cells represent the major source of CCL3 during -induced ileitis leading to the recruitment of CCR1^+^ inflammatory monocytes (Schulthess et al., 2012). However, IL-18 (and not IL-15) favored CCL3 secretion by these intestinal and pathogenic NK cells (Schulthess et al., 2012). The authors discussed the possibility that such pathogenic NK cells could cause the recruitment of inflammatory monocytes in Crohn’s disease (Schulthess et al., 2012). However, it is of note that the precise lineage of gut mucosa associated-NKp46^+^ cells remains debated and it is still unclear if they are developmentally related to the NK cells and/or to the lymphoid tissue inducer cells or if they represent a distinct innate immunity cell types (Colonna, 2009; Spits and Di Santo, 2011).

### HUMAN NK CELLS

In humans, the blood NK cell pool (CD19^−^CD3^−^CD56^+^ among lymphocytes) is commonly divided into two distinct subsets, based on the surface intensity of CD56 expression and the low-affinity Fc receptor CD16. A major population of CD56^dim^ NK cells (roughly 90%) expresses high levels of CD16, whereas a minor subset of CD56^bright^ NK cells fails to express CD16 (Lanier et al., 1986). Moreover, CD56^dim^ NK cells exert high levels of killer cell immunoglobulin-like receptors (KIR) and low levels of CD94/NKG2 receptors in contrast to CD56^bright^ NK cells (**Figure 1**). These two populations were shown to differ functionally in that CD16^low^ CD56^bright^ are considered more potent cytokine producers while CD16^high^ CD56^dim^ NK cells display higher cytotoxic functions. Only CD56^dim^ cells mediate CD16-dependent antibody-dependent cell-mediated cytotoxicity (ADCC; Cooper et al., 2001). However, although CD56^bright^ NK cells can produce high amounts of cytokine after 24 h stimulation with IL-12 or IL-18, CD56^dim^ cells also produce high quantities of cytokine after engagement of the activating receptors or after short-term cytokine stimulation (Fauriat et al., 2010; De Maria et al., 2011). This notion suggests that extrinsic factors (dictated by microenvironmental cues or soluble factors) may shape NK cell functions regardless of their “so called maturation stage.” Hence, CD56^dim^ and CD56^bright^ NK cells differ in their *in vivo* localization. CD56^bright^ NK cells express chemokine receptors (such as CCR7, CXCR3, and CD62L) favoring a preferential migration into lymphoid organs (LN) and a privileged interaction with LN-residing antigen-presenting cells (Campbell et al., 2001; Cooper et al., 2001). In contrast, CD56^dim^ NK cells are enriched in tonsils, lungs, mucosal sites, and the uterus (Ferlazzo et al., 2004). As a corollary to the murine data, CD27 has been shown to be expressed by the CD56^bright^ fraction of human NK cells and by LN resident NK cells in humans (Silva et al., 2008; Vossen et al., 2008). Analogous to the relationship between CD27^+^ and CD27^−^ mNK cells in mice, CD27^+^CD56^bright^ NK cells are considered as the immature subset of human circulating NK cells (Silva et al., 2008; Vossen et al., 2008).

Apart from blood and lymphoid tissues, organ-specific distribution and function of NK cells has been described. NK cells are present in normal liver and aid to control tolerance and homeostasis (Doherty and O’Farrelly, 2000), a dialog between Kupffer cells and NK cells allowing the tuning of hepatic NK cells during infection or liver injury (Tu et al., 2008). Half of hepatic NK cells are CD56^bright^ CD16^low^, express CCR7 and CXCR3, the inhibitory molecule NKG2A and high levels of TRAIL. Liver TRAIL-expressing NK cells contribute to hepatocellular damage and clearance of hepatitis C virus (Dunn et al., 2007; Stegmann et al., 2010). In the uterus (Whitelaw and Croy, 1996), *in situ* development of NK cells from precursors is induced by IL-15 and stem cell factor (SCF). NK cells can also be recruited by extravillous trophoblast (EVT) via CXCL12 secretion. Activated NK cells secrete IFN-γ that participate in the remodeling of spiral arteries. Decidual stromal cells secrete TGF-β that results in the down-regulation of CD16 expression, thus decreasing the ADCC of NK cells. Expression of HLA-C2 haplotype on EVT predisposes for preeclampsia (Hiby et al., 2004; Parham, 2004). In the mucosa surrounding the lymphoid follicles of tonsils and Peyer’s patches of the ileum/appendix, innate cells referred to as NK22 express the activating receptor NKp44 as well as the chemokine receptor CCR6 and may promote mucosal homeostasis (Cella et al., 2009). The expression of CC-chemokine receptor 8 (CCR8) and cutaneous leukocyte antigen (CLA) is restricted to human skin-resident NK cells. Skin NK cells are mostly CD56^+^/CD16^low^ and display strong cytotoxic activity against melanoma cells (Ebert et al., 2006).

Collectively, these data support the theory that the maturation stage of NK cells and environmental factors may cooperate to shape their functional activities, as already described for other innate cells such as DC. Although, in steady-state conditions, mNK cells can be found in some lymphoid and non-lymphoid organs, following an insult such as a infection, inflammation, or cancer, specialized NK cell subsets can be rapidly recruited to injured organs to perform their specific function (elimination of danger) and/or re-establish tissue integrity.

## NK CELLS ARE ABLE TO RECOGNIZE TUMOR CELLS

Tumor cells expose several ligands that can be recognized by NK cells rendering tumor susceptible to NK cell attack. Interestingly, accumulating evidences showed that NK cell-mediated elimination of tumor cells will lead to the subsequent development of tumor-specific T cell responses against the parental tumor cells (Diefenbach et al., 2001; Kelly et al., 2002).

### MOLECULES IMPLICATED IN THE RECOGNITION OF TUMORS BY NK CELLS

For more than 20 years, several lines of evidence demonstrated the important role of NK cells in the control of solid malignancies. The pioneering demonstration was conducted in the beige mouse in 1980 (Talmadge et al., 1980). Beige mice mimics human Chediak–Higashi syndrome presenting with defective natural cytotoxicity against tumor cells. Using a NK cell sensitive (but not resistant) tumor cell line, Talmadge et al. reported increased growth rate, faster induction time and increased metastatic dissemination of tumors established in beige compared to control mice. Later, depletion experiments using anti-asialoGM-1 or anti-NK1.1 antibodies concluded to the crucial role of mouse NK cells in keeping in check tumor growth and metastatic spread (Gorelik et al., 1982; Aboud et al., 1993; Smyth et al., 2001). Conversely, adoptive NK cell transfer could restore resistance to metastatic spreading, establishing a causal link between NK cells and tumor control in wild-type mice (Gorelik et al., 1982; Aboud et al., 1993; Smyth et al., 2000, 2001; Street et al., 2001). Moreover, a prominent role for Ly49 receptors (Ljunggren and Karre, 1985; Karre et al., 1986; Glas et al., 1992, 2000; Koh et al., 2001), NK group 2 member D (NKG2D) activating receptors (Diefenbach et al., 2001), DNAX accessory molecule-1 (DNAM-1; Lakshmikanth et al., 2009), and components of the NK cell secretory and cytotoxic machinery (Street et al., 2001; Takeda et al., 2011), TRAIL (Taieb et al., 2006) was reported in the control of tumor growth and metastases in mice.

In addition, translational research in patients argues on the role of NK cells in immunosurveillance against cancer (Imai et al., 2000; Guerra et al., 2008; Kreisel et al., 2012). The KIR (Moretta et al., 1996) and CD94/NKG2A heterodimers (Braud et al., 1998) participate in the regulation of NK cell activation by tumor cells. Indeed, tumor cells can down-regulate MHC class I expression the missing self-engagement of NK cell activation favoring tumor elimination (Vivier et al., 2012). Next to this mechanism, stress-induced molecules also activate NK cells. NKG2D plays an important role in the recognition of stress-induced molecules such as the polymorphic MHC class I chain-related molecules (MIC)A and MICB human (Groh et al., 1996; Bauer et al., 1999) and the cytomegalovirus UL-16 protein (ULBP; Cosman et al., 2001). Such proteins are frequently expressed in primary carcinomas (Pende et al., 2002; Vetter et al., 2002; Friese et al., 2003; Watson et al., 2006). The identification of two NKG2D haplotypes associated with differential NK cytotoxic potential and risk of cancer illustrates the importance of NKG2D in the anti-tumor immunosurveillance (Hayashi et al., 2006). Natural cytotoxicity receptors (NCR) are also implicated in the destruction of tumor cells. All three NCRs are implicated in the clearance of a variety of tumors, including carcinomas, melanomas, and neuroblastomas. Among self-ligands expressed by tumor cells and able to activate NK cells, heparan sulfate moieties of heparin sulfate proteoglycans have been described as self-modified ligands for NCRs (Jayson et al., 1998; Blackhall et al., 2001), these ligands being rarely expressed on normal cells (Bloushtain et al., 2004; Hershkovitz et al., 2007, 2008; Cagnano et al., 2008; Hecht et al., 2009; Jarahian et al., 2011). NKp30, NKp44, and NKp46 recognize highly sulfated heparan sulfate/heparin-type structures (Hecht et al., 2009). B7 homolog 6 (B7-H6), absent from normal cells but expressed on the K562 cell line and other acute myeloid leukemia and solid tumors, was identified as a ligand for NKp30. Moreover, nuclear factors (up-regulated or disturbed in their distribution) represent alternate ligands for NCRs. Hence, HLA-B-associated transcript 3 (BAT3) was identified as a ligand for NKp30 activating NK cells. BAT3 can be either re-localized at the cell surface or expressed on tumor-derived exosomes shed in the extracellular milieu (Pogge von Strandmann et al., 2007; Simhadri et al., 2008). Finally, proliferating cell nuclear antigen (PCNA) which is highly expressed in proliferating cells and associated with malignancy (Stoimenov and Helleday, 2009) was shown to be recruited in the immunological synapse formed between NK and tumor cells, leading to inhibitory triggering of NKp44 receptors (Rosental et al., 2011).

### NK CELLS CONSTRAIN METASTATIC DISSEMINATION

Several cell autonomous features resulting in NK cell recruitment and/or activation have been described. Hence, the interferon regulatory factor irf7 is a key molecule limiting bone metastases in a NK and CD8^+^ T cell-dependent manner (Bidwell et al., 2012). Enforcing irf7 expression by tumor cells could restore type I IFN signaling, leading to inhibition of metastatic dissemination in the 4T1.2 breast cancer bone metastasis model (Lelekakis et al., 1999; Eckhardt et al., 2005). Differences in gene expression profiles of tumor cells isolated from matched pairs of primary tumors and bone metastases revealed that a high number of IFN-related genes were down-regulated in bone metastases including irf7 (Bidwell et al., 2012). 4T1.2 clones overexpressing IRF7 were able to produce significant levels of IFN-α compared to the 4T1.2 control cell line. 4T1.2-irf7^+^ presented with reduced dissemination to the bones in an IFNAR1-dependent manner. Depletion of CD4^+^, CD8^+^, and NK cells demonstrated that CD8^+^ and NK cells (but not CD4^+^ T cells) were needed for the irf7-dependent anti-metastatic effect (Bidwell et al., 2012). Treatment with 10^5^ IU of recombinant IFN-α could reduce bone metastases in mice bearing the 4T1.2 tumors, causing the up-regulation of irf7, irf9, and STAT1 in the wild-type 4T1.2 cell line. Finally, authors showed that defective irf7 signaling pathway in human breast cancers was significantly associated with bone metastases as the first site of dissemination (Bidwell et al., 2012). Moreover, irf1 expression by the tumor was mandatory for NK cell-dependent suppression of metastases (Ksienzyk et al., 2011). Here, irf1 was implicated in the suppression of lung metastases, but not tumor spreading to the bone (Bidwell et al., 2012). Both type I and II IFNs were dispensable for the NK cell-mediated resistance to lung metastases. Irf1 expression by tumor cells was associated with an increased CXCL11/CXCR3-dependent NK cell infiltration of lung nodules. Moreover, irf1 could induce tumor cell surface expression of MHC class I molecules, death receptor DR5 and adhesion molecule CD155 (ligand for DNAM-1). Hence, NK cell-mediated elimination of lung metastases was mainly dependent on DNAM-1 receptors and only partially on TRAIL molecules (Ksienzyk et al., 2011). Altogether, these lines of evidence indicate the cell intrinsic role of interferon-related pathways in the control of metastatic dissemination by NK cells. Moreover, various subsets of NK cells appear to control bone (Bidwell et al., 2012) or lung (Ksienzyk et al., 2011) dissemination. Indeed, while CD27^+^ NK cells prevailed against bone marrow metastases (Bidwell et al., 2012), terminally differentiated NK cells seem to control lung metastases (Sceneay et al., 2012). Indeed, over 80% of lung NK cells are mature CD11b^+^ cells.

In contrast, other reports point to a role for myeloid cells in suppressing the function of mNK in metastatic niches. Sceneay et al. (2012) reported that hypoxia at the primary tumor site led to enhanced secretion of soluble factors that favor metastatic dissemination. Particularly, under hypoxic conditions, soluble factors secreted by the primary tumor induce the recruitment of bone marrow-derived cells in the lungs. Hypoxia caused the accumulation of granulocytic CD11b^+^/Ly6C^med^/Ly6G^+^ myeloid cells through a monocyte chemoattractant protein 1 (MCP1)-dependent mechanism as well as that of iNK cells (CD11b^−^/CD27^−^) endowed with poor anti-tumor effector functions. Authors assume that CD11b^+^/Ly6C^med^/Ly6G^+^ myeloid cells could inhibit NK cell differentiation, as myeloid cell accumulation has been inversely correlated with suppression of NK cell function in murine tumor models (Liu et al., 2007; Mauti et al., 2011). In methylcholanthrene-induced carcinogenesis as well as transplantable metastases models, NLRP3 inflammasome was shown to support metastases formation. Expansion of CD11b^+^ Gr1^int^ myeloid cells within the lung tumor microenvironment of NLRP3 knockout mice was concomitant with increased lung infiltrating activated NK cells and an improved anti-metastatic response. These myeloid cells secreted CCL5 and CXCL9 chemokines favoring the trafficking of NK cells into metastatic lungs (Chow et al., 2012).

Considering that NK cells can regulate metastatic dissemination, it remains unclear whether NK cells are able to modify the metastatic potential of primary tumor cells and premetastatic niche and/or could represent an efficient innate barrier that could prevent tumor cell implantation. To our knowledge, scarce data exist demonstrating that NK cells could modify tumor cells in the primary tumor in a way that would decrease metastatic potential. Some data suggested that NK cells could have cytotoxic activity against cancer stem cells, which represent a more chemo- and radio-resistant subpopulation within cancer (Tseng et al., 2010; Jewett and Tseng, 2011; Jewett et al., 2012). Considering accumulating evidence incriminating tumor stemness as a risk factor for metastatic relapse (van Zijl et al., 2009; Raimondi et al., 2010; Yu et al., 2012), high NK cell infiltration in the primary tumor could be a good biomarker associated with low risk of metastatic relapse. Recently, the epithelial-mesenchymal transition (EMT) process was linked to the gain of stem cell competence (Mani et al., 2008; Polyak and Weinberg, 2009). Thus, EMT favors the generation of tumor cells with self-renewing properties and high proliferative capacity, enhancing their chances to seed at a distant site and to grow metastases. Factors that can influence EMT and/or stemness such as hypoxia and TGF-β (Das et al., 2008; Heddleston et al., 2009) are also known to inhibit NK cell functions. Thus, it is conceivable that agents reinvigorating NK cells in the tumor bed could decrease its metastatic potential and thus reduce time to relapse.

In humans, NK cell infiltration (determined with different NK cell markers, i.e., CD57, CD56, NKp46, CD3, and CD56) is often studied on a tumor burden after curative resection. Interestingly, NK cell infiltrates are often associated with a lower risk of relapse and/or longer survival (**Table 1**). However, in most cases, NK cell numbers are limited compared with other effectors as reported using tissue microarrays of melanomas (Erdag et al., 2012; Sconocchia et al., 2012), hepatocellular carcinomas (Sconocchia et al., 2012), breast cancers (Sconocchia et al., 2012), renal cell carcinomas (Sconocchia et al., 2009), and colon rectal cancers (Sconocchia et al., 2010). Very low NK cell infiltration could be monitored in these different solid tumors, except for colorectal carcinomas where 38% of tumor samples appeared to be infiltrated by NK cells (Sconocchia et al., 2010, 2012). Furthermore, the activation status of NK cells infiltrating the tumor and/or tumor niches might play a crucial role in tumor clearance and/or prevention of tumor spreading. Many reports in cancer patients have demonstrated that NK cells have altered cytotoxic functions usually correlated with down-regulation of NCR while tumor cells usually express ligands for these NCR (Menard et al., 2009; Mamessier et al., 2011a,b; Platonova et al., 2011).

**Table 1:** Tumor infiltrating NK cells in human cancers.

SCancer	Stage	Sample size	Method	Markers used for NK cells analysis in IHC studies	Results	Reference
CRC	II–III	93	IHC	CD56, CD57	Low tumor stage (*P* = 0.004), marked CD8^+^ (*P* = 0.04, see Figure 2) and CD57^+^ (*P* = 0.05, see Figure 3) cell infiltration in the advancing tumor margin were correlated with a longer disease-free survival in multivariate analysis	Menon et al. (2004)
CRC	II–III	88	IHC	CD56	NK cells are rare compare to CD8^+^ cells (7/mm^2^ versus 76/mm^2^). Surprisingly in tumor with low expression of HLA class I higher CD8^+^, but not CD4^+^ cell infiltration was observed	Sandel et al. (2005)
CRC	IV	68	IHC	CD56	Thirty-three patients were treated with adjuvant FOLFIRI regimen or FOLFOX with Cetuximab and 35 patients received adjuvant FOLFIRI regimen or FOLFOX regimen alone. In cetuximab treated patients only CD56 and K-ras mutation status were independent predictors of the best overall response and progression-free survival. CD56^−^ tumors [HR 2.6 (95% CI: 1.14–6.00); *P* = 0.019] and K-ras mutations [HR 4.74 (95% CI: 1.8–12.3); *P* = 0.001] were significant independent negative prognostic markers for the PFS	Marechal et al. (2010)
CRC	IV	112	IHC	NKp46	Primary tumors and liver metastases were analyzed. Low NK cell number was detected in tumor and liver metastases. No correlation between NK cell infiltration and chemokine profile within the tumor tissue or relation between HLA class I expression and NK cell infiltrate were found. In this study no prognostic study was realized	Halama et al. (2011)
CRC	I–IV	1414	IHC (TMA)	CD56, CD57, CD16	This cohort included a mix of MMR deficient and proficient. CD56 cell infiltrate was observed in 38% of CRC. No prognostic role of NK cells infiltrate was determined in this cohort	Sconocchia et al. (2010)
Gastric CRC	I–IV	34 18	IHC, FCM	CD3, CD56	Patients with and without liver metastases were studied. The number of CD3^−^CD56^+^ (mostly CD56^dim^ NK cells) and CD3^+^CD56^+^ cells were decreased in metastatic livers compared to those unaffected by metastases. Interestingly lower CD56^+^ cell infiltration could be observed in patients with multiple liver metastases	Gulubova et al. (2009)
Gastric Esophageal	I–IV	50 35	IHC, FCM	CD56	NK cells inversely correlated with H_2_O_2_ production in the tumor microenvironment. The frequency of CD56^dim^ tumor-infiltrating NK cells decreased according to disease progression	Izawa et al. (2011)
GIST	IV	8	IHC, FCM	CD56	NK cells highly infiltrate GIST tumors. An inverse correlation was observed between NK cell infiltrate and metastasis occurrence.	Delahaye et al. (2011)
GIST	I–IIIB	47	IHC	CD56	Untreated c-Kit-positive primary GISTs were analyzed. NK cells represent a small fraction of tumor-infiltrating immune cells (34ŷ41 cells/mm^2^) compared to CD3^+^ cells (201ŷ331 cells/mm^2^) or monocyte-derived cells. No prognostic study was achieved	Cameron et al. (2008)
NSCLC	II–IIIA	150	IHC	CD57	In univariate analysis, CD57^+^, defined as CD57^high^, were associated with longer survival (*P* < 0.0002). CD57 cell infiltration is a prognostic factor in univariate analysis, but was not an independent prognostic factor from classical T and N classification in multivariate analysis	Takanami et al. (2001)
NSCLC	I–III	335	IHC (TMA)	CD56	Multivariate analysis showed that stromal CD56^+^ cells were an independent prognostic factor for disease-specific survival [HR 2.3 (95% CI: 1.1–5.0), *P* = 0.031]	Al-Shibli et al. (2009)
NSCLC	I–III	28	IHC (Frozen sections), FCM	NKp30, NKp46	NK cells infiltrating NSCLC are mostly CD56^bright^ NK cells with impaired cytotoxic capacities against tumor cells	Carrega et al. (2008)
NSCLC	I–II	86	IHC, FCM	NKp46	NK cells are recruited and localized in the stroma of the tumor rather than in the tumor nest. NK cells exhibited an altered phenotype with down-regulated NKp30, NKp80, CD16, NKG2D, and DNAM-1 while NKp44 and CD69 were over-expressed. Functional studies showed that tumor infiltrating NK cells had impaired cytotoxic functions compared to blood NK cells. Prognostic study in this cohort showed that the presence of NK cells is not associated with clinical outcome at early stages of the disease	Platonova et al. (2011)
Squamous cell lung carcinoma	IA–IIIA	50	IHC	CD57	Multivariate analysis including surgical-pathologic stage, age and endoscopy localization, the risk of death in patients with less than five CD57^+^ cell per field was 2.50 fold higher (95% CI: 1.07–5.85) than in those patients with more than five CD57^+^ cell per field	Villegas et al. (2002)
Prostate	Gleason 2–10	75	IHC	CD56	Forty patients were analyzed after radical prostatectomy and 35 after androgen deprivation therapy. In androgen deprivation treated patients, high number of CD56 cells was associated with a lower risk of prostate cancer progression (*P* = 0.044), while a high density of CD68 was related to an increased risk of biochemical recurrence (*P* = 0.011)	Gannon et al. (2009)
BC	I–III	204	IHC (TMA)	CD57	CX3CL1 expression correlates with CD8^+^ and CD57^+^ cell infiltrations. Tumor stage (I/II versus III), HER-2 status and CX3CL1 expression were independent prognostic factors for disease-free and overall survival	Park et al. (2012)
BC	I–III	140	IHC, FCM	CD56, CD3	In tumor tissue NK cells were enriched with CD56^b^^right^ cells with poor cytotoxic potential compared to normal mammary tissue. An inverse correlation between regulatory T cell and NK cell infiltrates was found. No prognostic study was achieved.	Mamessier et al. (2011b)
RCC	I–IV	117	IHC (TMA)	CD56, CD16	No CD56 cell infiltrates were detected in 92% of renal cell carcinomas	Sconocchia et al. (2009)
Melanoma	IIIA–IV	183	IHC (TMA)	CD56	Very low infiltration with CD56^+^ NK cells is described. CD4, CD8, and CD20 represented 80% of the immune infiltrate in this cohort	Erdag et al. (2012)
Melanoma HCC BC	ND	284 336 385	IHC (TMA)	CD56	No CD56 cell infiltrate was detected in 71.4% of melanomas, 92% of hepatocellular carcinomas and 97% of breast carcinomas	Sconocchia et al. (2012)

*CRC, colorectal cancer; NSCLC, non-small cell lung cancer; BC, breast carcinoma; GIST, gastro-intestinal stromal tumor; HCC, hepato-cellular carcinoma; RCC, renal cell carcinoma; ND, not described.*

**FIGURE 2:**
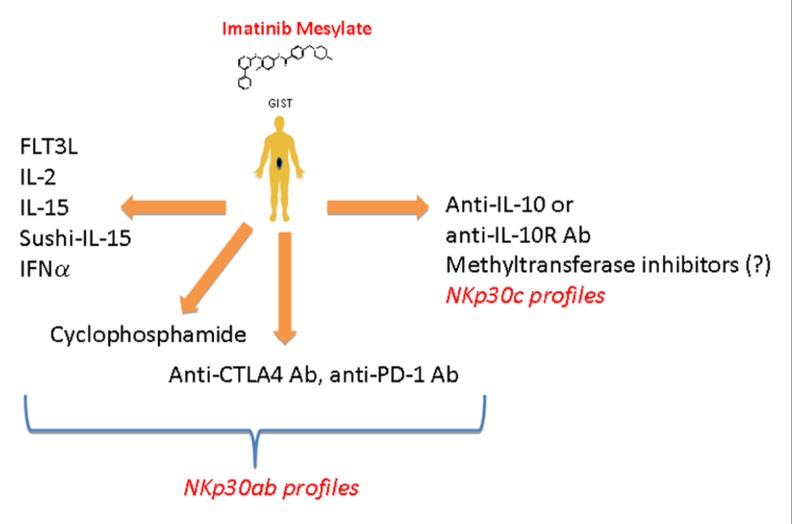
**Therapeutic strategies that could increase the functions of NK cells in patients with GIST**. NK cells of patients with high expression of NKp30a and/or NKp30b isoforms could be reinvigorated using treatments that are know to favor either direct activation (IL-2, IFN-α, IL-15, sushi-IL-15) or indirect activation (FLT3, cyclophosphamide) of NK cells along with imatinib mesylate treatment. Since Balachandran et al. (2011) have shown that CD8^+^ T cells could favor tumor regression it is conceivable to use drugs that will favor CD8 T cell activation (anti-PD1 or anti-CTLA4). In patients harboring the wrong NKp30 profile (high expression of NKp30c isoform) blocking IL-10 or IL-10 receptor (IL-10R) or using agent that could modify the NKp30 profile toward the NKp30ab profile (methyltransferase inhibitors?) might restore the NKp30-dependant NK cells function, thus favoring the control of tumor growth and/or dissemination.

Over the past decade, we investigated the role of NK cells in the control of gastrointestinal sarcomas (GIST). Unexpected long-term responses to imatinib mesylate (IM) have been reported in GIST lacking the hallmark molecular criteria of responses to IM (Borg et al., 2004; Menard et al., 2009), suggesting that IM might mediate part of its therapeutic effect by an off-target effect. Indeed, several mouse tumor cell lines that did not respond to IM *in vitro* did so *in vivo* in immunocompetent mice. This therapeutic off-target effect of IM was mediated by NK cells and/or specialized DC subsets (Borg et al., 2004). We found that, by inhibiting c-Kit in DC, IM could promote a DC/NK cross-talk that ultimately stimulates NK cells to produce IFN-γ both in mice and in humans (Borg et al., 2004). Importantly, the IM-induced IFN-γ production by NK cells represented an independent predictor of long-term survival in advanced GIST treated with IM (Menard et al., 2009). Since IM failed to trigger NK cell IFN-γ production in about half of GIST patients, we launched a comprehensive analysis of the GIST-associated NK cell phenotype. At diagnosis, circulating NK cells from GIST patients (compared with healthy volunteers) exhibited a selective down-regulation of one particular type of stimulatory NK cell receptor, NKp30, but not that of another type, NKG2D, and this NKp30 down-regulation was only partially restored by IM (Menard et al., 2009). We demonstrated that the alternative splicing of exon 4, which affects the intracellular domain of NKp30, generates three membrane-bound proteins with distinct functions. Indeed, the NKp30a isoform could stimulate NK cell degranulation and Th1-type cytokine secretion, while the NKp30b isoform only signals for Th1 cytokine secretion and the NKp30c isoform transduced a delayed signal leading to IL-10 secretion. In a cohort of 80 patients with metastatic or recurrent GIST, the predominant expression of NKp30c was associated with decreased NKp30-dependent TNFα and CD107a release, compared to patients with high expression of NKp30a and/or NKp30b. The predominant expression of the NKp30c isoform was an independent prognostic factor of reduced overall survival (Delahaye et al., 2011). Freshly dissociated tumor samples revealed that GIST are highly infiltrated with NK cells (roughly 25% of CD45^+^ leukocytes were CD3^−^CD56^+^ NK cells), and the density of NK cell infiltration was inversely correlated with metastases at diagnosis (Delahaye et al., 2011). Altogether, these data again support a more preventive than curative role for NK cells by counteracting metastatic spread. Since NK cells could play a crucial role in controlling tumor dissemination in GIST patients, we developed strategies for boosting NK cells along with IM treatment. Association of IM and IL-2 has been shown to have potent anti-tumor responses in mice through activation and trafficking of IKDC to lung metastases (Taieb et al., 2006). From 2009 to 2012, we launched a phase I clinical trial in patients with refractory solid tumors to determine the maximum tolerated dose of IL-2 combined with 400 mg daily IM. Data from this clinical trial will be reported soon. If the association appears to be non-toxic, a phase II study in GIST patients combining these two compounds will be considered. Other NK cell stimulatory compounds to be used in synergy with IM against GIST could be envisaged (**Figure 2**).

Thereby, it seems that NK cells could be a potent effector cells preventing tumor formation (immunosurveillance) and/or limiting metastatic spread. Previous study by Imai et al. (2000) had anticipated this type of conclusion in humans. This 11-year follow-up study showed a reduced cancer risk among people with strong or medium natural cytotoxic functions in peripheral blood, suggesting a role for natural immunological host defense mechanisms against cancer. However, and as described above, NK cells have difficulty entering the tumor therefore, strategies that could increase the penetration and/or proliferation and/or local differentiation of NK cells in the tumor remain attractive. Recently, the B16F10 transplantable mouse melanoma model transfected with chemerin, a chemoattractant for NK cells, macrophages, and DC subsets (Wittamer et al., 2003; Zabel et al., 2005; Parolini et al., 2007), led to a significant recruitment of NK cells in the tumor responsible for tumor regression (Pachynski et al., 2012). The authors showed that intra-tumor injection of the recombinant chemerin was sufficient to obtain tumor regression. Finally, in two independent clinical cohorts of melanoma patients, retention of high RARRES2 (a gene encoding the chemoattractant chemerin) expression correlated with better clinical outcomes (Pachynski et al., 2012).

## CONCLUDING REMARKS

We are entering a new era of NK cell investigations in human malignancies that will open up new avenues for NK cell identification and profiling. How NK cell trafficking to tumors and/or differentiate from precursors in tumors remain an active conundrum. How NK cells prevent metastases is obscure. How NK cells regulate adaptive immunity is still debated since NK can shut down or instead trigger DC or CD4^+^ T cells. How to strengthen the functions of NK cells and/or neutralize the immunosuppressive factors should be harnessed (e.g., IL-2, IL-15, sushi IL-15, IFN-α, and peptide inhibitors of TGF-β1; **Figure 2**).

## Conflict of Interest Statement

The authors declare that the research was conducted in the absence of any commercial or financial relationships that could be construed as a potential conflict of interest.

## Abbreviations

AM: alveolar macrophages
CCL2: CC-chemokine ligand 2
CCL5: CC-chemokine ligand 5
CCR2: CC-chemokine receptor 2
CCR5: CC-chemokine receptor 5
CCR8: CC-chemokine receptor 8
CD: cluster of differentiation
CLA: cutaneous leukocyte antigen
CSF1R: colony stimulating factor 1 receptor
CX3CL1: CX3C-chemokine ligand 1
CX3CR1: CX3C-chemokine receptor 1
CXCL10: CXC-chemokine ligand 10
CXCL11: chemokine (C-X-C motif) ligand 11
CXCL12: CXC-chemokine ligand 12
CXCR3: CXC-chemokine receptor 3
CXCR6: CXC-chemokine receptor 6
DC: dendritic cell
DSC: decidual stromal cells
EVT: extravillous trophoblast
GIST: gastrointestinal stromal tumor
HCV: hepatitis C virus
HLA: human leukocyte antigen
IBD: intestinal bowel diseases
IFN-α: interferon-α
IFN-γ: interferon-γ
IFNAR1: interferon (alpha beta omega) receptor 1
IL: interleukin
IM: imatinib mesylate
IP-10: interferon gamma-induced protein 10
KIR: killer cell Ig-like receptor
KLRG1: killer cell lectin-like receptor subfamily G member 1
MCSF: macrophage colony-stimulating factor
NCR: natural cytotoxicity receptors
NK cells: natural killer cells
PD-1: programmed cell death protein-1
PD-L1: programmed cell death 1 ligand 1
RANK: receptor activator of nuclear factor κB
RANKL: receptor activator of nuclear factor κ-B ligand
SA: spiral arteries
TRAIL: TNF-related apoptosis-inducing ligand
T_reg_: regulatory T cell.
